# Data on dental bite materials with stability and displacement under load

**DOI:** 10.6026/973206300161145

**Published:** 2020-12-31

**Authors:** Harsh Kasabwala, Subhabrata Maiti, V Ashok, Keerthi Sashank

**Affiliations:** 1 Saveetha Dental College, Saveetha Institute of Medical and Technical Sciences, Saveetha University, Chennai 77, India

## Abstract

It is of interest to document the accuracy, time dependent dimensional stability and displacement to load for 5 types of inter-occlusal recording materials. We used cad bite paste, putty, lab putty, pattern resin and poly tray acrylic material in this study. A total of 25 samples were fabricated to evaluate the accuracy and dimensional stability. The amount of displacement was calculated by applying displacing force on the bite registration materials till they fractured using Instron 3000 UTS machine. Statistical analysis of the data shows that CAD bite paste has the maximum amount of accuracy and dimensional stability whereas pattern resin showed the least amount of displacement to applied force. Thus, we report that CAD bite registration material displayed best results in terms of accuracy and dimensional stability and moderate results in terms of displacement to force.

## Background

Phillip Pfaff was the first to record interocclusal relations using natural waxes in 1756 [2,3,4,5]. This led to the development of various different bite materials and other impression materials that have been modified to give better handling characteristics. Impression plaster, model plaster, poly vinyl siloxane, pattern resins etc can be used as interocclusal recording materials [6], [7], [8]. ZOE and waxes were initially used in bite registration, they are still used quite frequently due to their low cost and ease of availability [9,10]. Currently with the development of elastomeric materials, the practice of using waxes and other old materials is decreasing. The ideal prerequisite for fabrication of any prosthesis is accurate articulation of the casts [1,11]. Prosthesis can be called clinically acceptable when it is in harmony with the stomatognathic system. Any type of restoration can be deemed successful only when it can replicate or transfer the accurate interocclusal record. There are mainly 3 reasons for development of inaccuracies while recording interocclusal record [9] (1) the biologic characteristics of stomatognathic system, (2) Manipulation of the material, and (3) The properties of the interocclusal recording materials. The manner in which the dentist handles the material in different clinical phases and scenarios, will decide the accuracy and dimensional stability of the material. Delay in carrying materials to distant laboratories is the most common factor leading to dimensional changes. The mounting is delayed and by that time the interocclusal material might have minor changes in their dimension causing improper articulation. Additionally materials having a great amount of dispersibility tend to be very flexible and have a great amount of bounce back. This might also affect the final articulation and recording of accurate jaw relation. Thus in order to recording an accurate jaw relation and to avoid error during articulation procedure it is necessary for the inter occlusal relation recording material should have good accuracy, dimensional stability and moderate rigidity [12]. Chemically these elastomeric bite registration materials are similar to the impression materials that have been used for many years [13,14,15,16]. Plasticizers and various catalyzing agents have been added in order to improve their chemical and physical properties [17]. Therefore, it is of interest to document the accuracy, time dependent dimensional stability and displacement to load for 5 types of inter-occlusal recording materials.

## Materials and Methods:

The ethical approval for this study was received from the Ethical research committee SIMATS Chennai. Tooth preparation was done for maxillary right first molar and first premolar in a typodont set. Three pin holes were notched on the mesiopalatal, mesiobuccal and distobuccal cusp tips of the prepared acrylic first molar which acted as reference points to measure the buccolingual and mesio distal distance. Slight downward finger pressure was used while recording the impression. After the setting of the material the bite registrations were immediately poured using dental gypsum type III orthokal material (Kalabhai Karson, India). This material exhibits excellent surface and brilliant details. It provides adequate working time and good dimensional stability. All the manufacturers instructions were followed while mixing and pouring the material to avoid irregularities and dimensional changes. After the setting of the poured dies, they were carefully retrieved. The three notches made on the acrylic molar were replicated in the orthokal die and the mesio distal and labiolingual dimensions were measured using a digital vernier calliper. This helped in recording the accuracy of the material. The same procedure of pouring the bite records was repeated at an interval of 24 hours, 48 hours and 72 hours. The samples were then stored in moisture free polyethylene bags at room temperature of 28±2°C in between testing hours. This was done in order to evaluate dimensional stability of the material. SPSS software version 20 was used to carry out the statistical analysis. Inter sample evaluation was done using Mann Whitney test whereas Intra sample evaluation was done using one way ANOVA test. The displacement to force was evaluated by making a metal template. The bite registration materials were poured and the mould was made. The centre of the mould was marked and a displacing force was applied on the moulds using a universal testing machine [INSTRON 3000 series]. The displacing force was applied till the mould fractured. The amount of force in kN, the flexural stress at maximum force and the amount of displacement was recorded.

## Results:

The accuracy and dimensional stability values were maximum in case of CAD bite bite registration paste followed by putty, lab putty, pattern resin and poly tray material respectively in both the directions mesio distally as well as bucco lingually (Table 1, Table 2 and Table 3 - see PDF). Post Hoc analysis also showed that CAD bite had superior dimensional stability values. There was a significant difference between the dimensional stability of all three materials at different intervals with P-value <0.05 in both the directions mesio distally as well as bucco lingually. Comparatively the CAD bite registration paste showed less distortion with good dimensional stability as compared to the remaining materials at 1 hour, 24, 48, and 72 hours. The maximum displacement to force was seen in case of putty material suggesting that it will have the maximum bounce back during mounting of casts followed by CAD bite paste, lab putty, pattern resin and poly tray material. Pattern resin and poly tray had significantly lower values as compared to the other three materials. P value after NOVA test was 0.05 suggesting that there was a significant difference between those values of all the bite registration materials in terms of displacement in mm. The maximum force that the material could withstand in N was maximum for pattern resin, followed by poly tray material, CAD bite paste, putty and lab putty respectively. The P value for the maximum force the materials could withstand was less than 0.05 suggesting that there was a significant difference in the ability of the different bite materials to withstand force subjected on them (Figure 1 - see PDF).

## Discussion:

After the introduction of different inter occlusal recording materials it is always difficult to decide as to which material should be used in routine clinical practice for precise recording and transferring of accurate existing occlusal records. This will help in the accurate articulation of patient's diagnostic or working casts. This will intern help in fabricating a good and satisfactory prosthesis. Hence, the present in-vitro study was carried out in order to evaluate the accuracy, the time dependent dimensional stability of the bite materials at 24, 42 and 72 hours and the amount of displacement that the material would undergo when subjected to maximum force. Operator skills are the paramount while manipulating the materials before recording the bite registration [1]. Zinc Oxide eugenol is a traditional bite registration paste. It is easy to manipulate and is pocket friendly [18,19,20]. It wasn't included in this study due to the irritation it causes to the tissues as well as the poor results shown in the study by [1]. CAD bite registration paste showed the most superior amount of accuracy and dimensional stability suggesting that the articulation of the casts can be delayed by a day or two if the dental laboratory is situated at a distance and will require a couple of days for the bite record to reach there. Poly tray material on the other hand showed the least accuracy as well as dimensional stability values suggesting that it would not act as an ideal bite registration material. Generally the mounting of the casts is not done in the clinical setup hence it is necessary for the bite recording material to be dimensional stable so that the record does not distort till the mounting is done in a laboratory setup. In terms of amount of is placement to force undergone by any material, putty material showed the maximum amount of displacement in mm with a mean and standard deviation value of 9.78 ± 0.20 mm. Poly tray material on the other had had the least displacement values suggesting that it was very rigid. Despite its superior rigidity poly tray material can be excluded from the list of ideal bite registration materials due to its poor dimensional stability and brittleness. Putty material having good dimensional stability had very high values of displacement suggesting that it will have a lot of bounce back while mounting the dental casts which might create inaccuracies during mounting. Lab putty had satisfactory values for displacement to force and its dimensional stability values were also average suggesting that it can be used as a bite registration material. Pattern resin had poor dimensional stability values suggesting that it might lead to inaccurate articulation if the mounting is delayed. On the other hand it had the least displacement values suggesting that it was very stable and rigid. It also showed the maximum value in the ability of withstanding force. There are no studies that have been done that compare the bite registration materials used in this particular study in terms of accuracy, dimensional stability and displacement to applied force.

## Conclusion:

We report that CAD bite registration material displayed best results in terms of accuracy and dimensional stability and moderate results in terms of displacement to force.

## Author contributions:

Author 1 Harsh Kasabwala carried out the study by collecting the raw data handwriting the manuscript with the necessary statistical analysis. Author 2 Dr Shubhabrata Maiti helped in guiding the study and supervised the statistics.
